# The Prevalence of Dental Carious Lesions and Associated Risk Factors in Chinese Children Aged 7-9 Years

**DOI:** 10.3290/j.ohpd.b5628793

**Published:** 2024-07-26

**Authors:** Hui Li, Xiaoyu Liu, Jianhui Xu, Siwei Li, Xin Li

**Affiliations:** a Dentist, The Second Affiliated Hospital of Jinzhou Medical University, Jinzhou, China. Participated in research design, wrote research manuals, conducted statistical evaluation, and carried out data interpretation and analysis.; b Dentist, The Second Affiliated Hospital of Jinzhou Medical University, Jinzhou, China. Collected data.; c Dentist, The Second Affiliated Hospital of Jinzhou Medical University, Jinzhou, China. Proofread the manuscript, data curation.; d Professor, Shenyang Medical College, Shenyang, China. Provided critical comment on the design, manuscript, and data analysis.; All authors read and approved the final manuscript for publication.

**Keywords:** carious lesions, children, prevalence, risk factors

## Abstract

**Purpose:**

To investigate the prevalence, severity, oral distribution, and associated risk factors of carious lesions in the primary teeth in children in Jinzhou, China, aged 7-9 years.

**Materials and Methods:**

A total of 1603 primary school students aged 7-9 years old from public and private schools in Jinzhou were recruited using multi-stage, stratified, and random sampling methods for cross-sectional studies. Carious lesions in the primary teeth of school-age children were detected and recorded according to the World Health Organization standard, and a questionnaire was collected from a parent or guardian with information on the relevant risk factors for the child. Odds ratios and 95% confidence intervals of factors related to carious lesions were estimated using binary logistic regression analysis (p<0.05).

**Results:**

The prevalence of carious lesions in the primary teeth was 74.5%, the average number of carious lesions was 3.02, and dmft was 4.08 ± 2.74. There were 655 cases (77.1%) of dental carious lesions in boys and 546 cases (72.5%) in girls, and the difference between them was statistically significant (p<0.05). Binary logistic regression analysis showed that the mother’s educational level, brushing frequency, brushing time, and consumption of soft drinks, desserts, and sweets were all associated with a higher prevalence of carious lesions (p<0.05).

**Conclusions:**

The children in our sample had a high incidence of carious lesions of the primary teeth, especially the mandibular primary molars. Social demographic factors, oral hygiene habits, and dietary habits all play an important role in the occurrence of carious lesions.

Dental caries is a very common oral disease that can cause significant psychological, physical, and social trauma in children.^14,17,34^ Globally, untreated caries affects 3.9 billion people, 60% to 90% of whom are school-age children.^14,15^ The results of two epidemiological surveys in China in 1995 and 2005 showed that the rate of caries in the primary teeth of 5-year-old children increased from 66% to 70.9% during this time and that the average dmft increased from 2.71 to 4.23.^37^ The reason for this upward trend has been found to be related to the increase of people’s purchasing power due to China’s economic growth during this timeframe, which led to a large increase in sugar consumption without a corresponding increase in demand for oral health care.^13,23^

There are many aetiological aspects to of caries, including oral hygiene, dental service utilisation, environmental factors and other modifiable risk factors, as well as genetics, dental morphology, and other immutable risk factors that jointly induce diseases.^14,15,33^ However, due to cultural differences, dietary patterns, and other factors in different countries and within each country, people’s daily living habits and lifestyles vary, so that oral behaviour, caries incidence, and related government policies are all different as well.^18,33^

Schoolchildren aged 7-9 years old are in the early stages of dental replacement, which is the best time to prevent caries. With the growth of the dental arch, primary teeth fall out and permanent teeth erupt, causing temporary crowding and uneven arrangement of teeth, which can cause food impaction, leading to a poor oral hygiene environment that allows diseases such caries to flourish. In addition, children at this stage have just entered school and are still experiencing rapid cognitive development. This is a key period for instilling oral health knowledge and forming good behavioural habits.^4^ Therefore, regular oral-health epidemiological surveys should be carried out in special populations to obtain the most useful oral health information to help with the planning and evaluation of health-care programs as well as conductin targeted intervention measures to prevent caries, such as dissemination of oral health-care knowledge and provision of pit-and-fissure sealants.^25^

The caries prevalence among school-age children is high in China. The latest survey in Liaoning Province was in 2020, and the survey population was 3- to 5-year-old preschool children.^38^ Studies on mixed dentition in school-age children in northern China, especially in northeast China, are still very limited. In Jinzhou, Liaoning Province, China, a major transportation hub connecting north China and northeast China, there is a diversity of ethnic groups, and at present, investigations on caries in school-age children in this region are lacking.

Caries, the most common oral-health problem in school-age children, is a progressive, destructive disease caused by long-term interactions between microorganisms, substrates, and teeth. Therefore, the purpose of this study was to evaluate the prevalence, severity, oral distribution, and related factors of decayed teeth in schoolchildren aged 7-9 years in Jinzhou City, China, to provide a scientific basis for the government and public-health decision-makers to formulate oral disease prevention and control programs and related policies.

## MATERIALS AND METHODS

### Ethics Approval and Consent to Participate

The study protocol was designed in compliance with the Declaration of Helsinki, and the study was approved by the Ethics Committee of the Second Affiliated Hospital of Jinzhou Medical University. Written informed consent for children to participate was obtained from their parents or guardians prior to data collection. The project name was “Regulation of autophagy by F90/circBPTF/miR-762/PIK3R5 pathway in pulmonary epithelial cells infected by Porphyromonas gingival combined with HINI virus”, item number: 2023JH2/101300079; project source: Applied Basic research program of Science and Technology Department of Liaoning Province.

### Study Design and Sample

Based on a cross-sectional study from China, the expected prevalence was 68%.^13^ In our study, the accuracy level was 10%, the confidence interval (CI) was 95%, and the sample size was increased by 10% to take into account truancy and exclusion criteria. Therefore, 1647 students were required for this study. Random sampling was used to select six local primary schools: Fulun Primary School, Guohe Primary School, Yuying Primary School, Experimental Primary School, Railway Primary school, and Dabei Primary School. The inclusion criteria were (1) signed parental informed consent and agreement to participate in the study; (2) having resided locally for more than 6 months as of the survey date; (3) aged 7-9 years. The exclusion criteria comprised (1) congenital oral disease; (2) refusal to cooperate with the oral examination even after persuasion attempts; (3) children undergoing orthodontic treatment; (4) missing questionnaire items making up ≥1/3 of the total or the same answer was given to all questions.

### Clinical Examination and Questionnaire

This survey was conducted in an empty classroom with all teeth examined using portable light sources, disposable oral examination instruments (oral scopes, oral probes, gloves, and cotton swabs), and community periodontal probes (CPI). According to the epidemiological investigation methods and standards for carious lesions recommended by the WHO,^36^ cotton swabs and probes were used to remove food residue on the tooth surface before examination. Carious lesions were diagnosed as marked cavitations or marked subenamel destruction in the approximal spaces or smooth surface of the tooth, or apparent lesions on the bottom or wall of a cavity which proved soft upon testing with a dental probe. During the examination, if there was any doubt, coronal carious lesions were not recorded as being present. The possible categories of missing primary teeth (mt) due to caries were as follows: loss of teeth that should not have fallen out, such as primary canines and primary molars, and carious lesions that occurred prior to tooth extraction. dmft (decayed, missing, and filled primary teeth) were the classifications used to evaluate the status of primary teeth coronal carious lesions in our sample. dmft > 0 indicates presence of carious lesions, and dmft ≤ 0 indicates absence of carious lesions.

After the completion of the clinical examination, the parents or guardians of the participants completed a questionnaire on the risk factors associated with caries based on the fourth National Oral Health Epidemiological Survey, including social demographic factors (gender; age; area; mother’s educational level as either low: primary, moderate: secondary or high school, or high: university), oral hygiene habits (brushing frequency; brushing time; flossing or not), and dietary factors (soft drinks: juice/cola, etc; desserts: cookies/cake, etc; confections [candy]).

### Statistical Analysis

Statistical analysis was carried using SPSS software, version 25.0 (IBM; Armonk, NY, USA). All variables were expressed as percentages and mean ± standard deviation. Comparison of caries prevalence was made using chi-squared tests, and based on these results, statistically significant variables were included in binary logistic regression analysis. The odds ratios (OR) and their corresponding 95% CIs were then calculated to distinguish risk factors. Finally, α = 0.05 for all tests and p<0.05 were considered to indicate statistically significantly different test results.

### Reliability Test

There were 3 inspectors and 3 recorders in this survey, all of whom were medically-licensed dental professionals and had more than two years of dental work experience. In the early stage of the investigation, all inspectors and recorders also received uniform theoretical and clinical knowledge training. Before the final investigation, 20 students were randomly selected for repeated testing to evaluate inter- and intra-examiner agreement. Kappa coefficients for intra-examiner aggreement for the 3 inspectors were 0.92, 0.90, and 0.87, and the kappa coefficient for inter-examiner agreement between all three inspectors was 0.80. During their formal investigation, each inspector randomly selected 5% of all subjects each day for a secondary test, with kappa coefficient values > 0.85.

## RESULTS

### Participants’ Information

Among the 1647 participants, 18 subjects did not meet the inclusion criteria and were excluded, 20 subjects lacked items or missed ≥1/3 of the items on the questionnaire, and 6 students’ parents did not sign the informed consent form. Thus, 1603 participants underwent a complete clinical examination and the questionnaire survey, yielding a participation rate of 97.3%. The survey revealed that 1194 students had carious lesions (dmft > 0 in at least one tooth). Among them, 546 were girls and 655 were boys. The overall caries prevalence was 74.5%, the average number of dental carious lesions was 3.02, and dmft was 4.08±2.74.

### Distribution and Quantity of Carious Lesions

**Fig 1:**
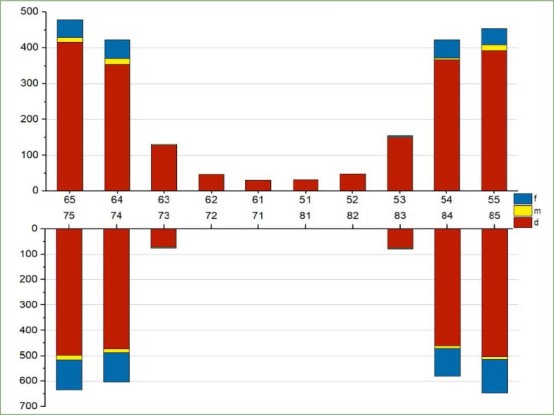
The prevalence of carious lesions in different primary teeth positions.

Figure 1 shows the distribution of carious lesions at different dental positions. A total of 4849 carious lesions were found in the 1603 participants. Unfilled teeth with carious lesions accounted for 84% of the total, 0.2% of teeth with carious lesions had been lost, and 1.4% of the carious lesions occurred in filled teeth. In the anterior dentition, the prevalence of carious lesions in the maxillary teeth was statistically significantly higher than that in the mandibular teeth; the mandibular anterior teeth had the lowest caries prevalence. In the posterior dentition, the prevalence of carious lesions in the mandibular teeth was higher than in the maxillary teeth and was especially prevalent in the mandibular second primary molars.

**Fig 2:**
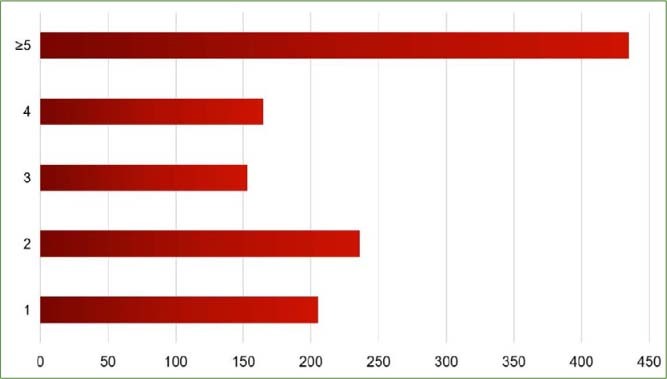
The frequency distribution of carious lesions in primary teeth.

Figure 2 shows the number of carious lesions in the mouth of each child. 37% of the children had 1-2 carious lesions, and 63% had more than 3. Strikingly, the number of carious lesions was more than 5 in 36.4% of children. In most cases, the carious lesions were symmetrically distributed on the right and left sides of the mouth.

### Risk Factors for Caries Prevalence

Table 1 shows the frequency distribution of social demographic variables; the prevalence of caries decreases with age, with statistically significant differences by age, sex, and mother’s educational level (p<0.05). Table 2 shows the frequency distribution of other influencing factors. Here we see that children with shorter brushing frequency and duration, and higher intake of sugary foods have a higher incidence of caries (p<0.05).

**Table 1:** Frequency distribution of social demographic variables

Variables	N (N=1603)	Carious lesions	X^2^	p-value
		dmft>0 n (%)		
**Gender**				
Female	753	546 (72.5%)	4.397	0.036
Male	850	655 (77.1%)		
**Age in years**				
7	532	428 (80.5%)	17.189	0.000
8	523	393 (75.1%)		
9	548	381 (69.5%)		
**Residential area**				
Urban	1224	934 (76.3%)	1.139	0.286
Rural	379	279 (73.6%)		
**Mother’s educational level**				
Low (primary)	627	469 (74.8%)	104.275	0.000
Moderate (secondary, high school)	314	208 (66.2%)		
High (university)	662	315 (47.6%)		


**Table 2:** The relationship between various variables and the prevalence of dental carious lesions

Variables	N (N=1603)	Carious lesions	X^2^	p-value
		dmft>0 n (%)		
**Brushing frequency**				
<2/day	982	756 (77.0%)	77.860	0.000
≥2/day	621	348 (56.0%)		
**Brushing time**				
<2 min	957	741 (77.4%)	109.241	0.000
2-3 min	646	339 (52.5%)		
**Flossed**			56.003	
Yes	621	313 (50.4%)		0.000
No	982	678 (69.0%)		
**Frequency of soft drink consumption**				
≥2 times/day	256	202 (78.9%)		
≥2 times/week	569	377 (66.3%)		
<2 times/week	778	342 (44.0%)	124.196	0.000
**Frequency of dessert consumption**				
≥2 times/day	213	167 (78.4%)		
≥2 times/week	457	297 (65.0%)		
<2 times/week	933	404 (43.3%)	116.320	0.000
**Frequency of sweets consumption**				
≥2 times/day	472	389 (82.4%)		
≥2 times/week	397	279 (70.3%)		
<2 times/week	734	312 (42.5%)	211.123	0.000


**Fig 3:**
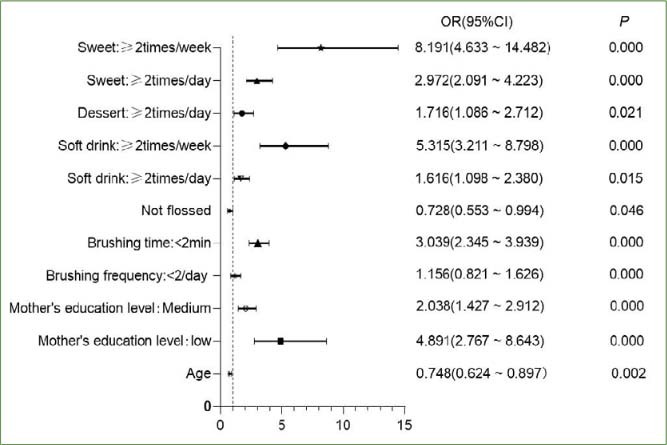
Risk factors using binary logistic regression.

The variables with statistical significance (chi-squared) in Tables 1 and 2 (p<0.05) were included in a binary logistic regression analysis (Fig 3), and the risk factors and protective factors were identified with OR=1 as the standard. In addition to age and using dental floss (OR<1; p > 0.05), other variables can be considered independent risk factors for caries (OR>1; p<0.05) as well.

The datasets are available from the corresponding author on reasonable request.

## DISCUSSION

Our epidemiological survey revealed that the caries prevalence rate was high among children aged 7-9 years in Jinzhou City. Carious lesions were found in 1194 of 1603 students (dmft >0). We found that tooth decay is more likely to occur in certain susceptible teeth, but not all teeth. Molars are more prone to decay than are incisors, premolars, and canines, and mandibular molars are the most caries-prone. Furthermore, mandibular incisors and premolars/canines are the least prone to caries, which is consistent with literature.^25,27^ This is because mandibular teeth generally erupt before maxillary teeth and are therefore exposed in the mouth for a longer period of time. Second, related to the specific macromorphology of the occlusal surface of molars, there are many irregular, narrow, and deep pits and fissures. In the pits and fissures, the enamel thickness is often insufficient. Furthermore, because molars are located far to the posterior in the dental arch, they are difficult to clean, which favours plaque growth.^5,20^ Finally, the mandibular anterior teeth are located at the opening of salivary glands such as the submandibular gland. Saliva provides lubrication and mechanical cleaning for the mouth so that dietary sugars and acids produced by plaque metabolism are flushed away after being exposed to carbohydrates. This effectively controls the pH of the biofilm and prevents enamel demineralisation.^26^ In addition, thanks to their position in the mouth, the mandibular front teeth are easier to clean.

In this study, the prevalence of caries in primary teeth in children aged 7-9 years in Jinzhou was higher than that in Wuhan (67.7%)^13^ and Guangzhou (30.7%).^22^ The Wuhan and Guangzhou studies were consistent with the diagnostic criteria used in this study, but, due to the different age groups they encompassed, differences in the epidemiological investigations, different clinical examination methods and diagnostic criteria, including dmfs and ICDAS, the results cannot be directly compared.

According to the report Chinese Students and Health Study in 2005, the prevalence of caries in the primary teeth and dmft of 5-year-old children in Liaoning Province were 73.86% and 4.38,respectively, both of which were lower than those in 1995 (89.5% and 6.8, respectively).^37^ However, compared to our study, the caries incidence in Jinzhou was slightly higher than that in Liaoning province (74.4% vs 73.9%), possibly because the one-child policy implemented by the Chinese government had a greater impact in the northeast region, leading to more only-children whose parent(s) may have been more inclined to indulge them. Second, most of the children in our sample live in cities, where socioeconomic status levels are slightly higher than in rural areas, and it was easier for them to obtain carbohydrates in their diet. Finally, these results may also have been closely related to the low level of oral health care for children.

Regarding the sociodemographic variables, our survey revealed that the caries prevalence in girls and boys was 72.5% and 77.1%, respectively, with a statistically significant difference (p<0.05) between the two groups, which is consistent with the results of several previous studies.^1,11,19,22,35^ This difference may be attributed to the fact that girls tend to pay more attention to the appearance of their bodies and teeth, have lower self-esteem than boys, are more sensitive to oral diseases, and are impacted more by all these aspects in terms of quality of life.^7,19^ As a result, girls tend to have a more positive attitude towards oral health, a healthier lifestyle, and a better level of oral hygiene.

Our results were consistent with several existing studies for age as well,^1,32^ especially for our finding that the carie rate of primary teeth decreased with age, which is related to primary-tooth loss and permanent-tooth eruption. In terms of household registration types, we found that the caries prevalence among children in urban families was higher than that in rural families, but the difference was not statistically significant (p>0.05). This is consistent with the literature;^11,29^ and the reason for the difference could be differences in age groups and the fact that most of the children in our study came from urban areas. The second is the changing diet structure in the Northeast, which has made sugary processed foods more accessible to urban residents, and reduced consumption of whole grains.

In terms of educational attainment, a study in 2020^6^ showed that mothers pay more attention than fathers to their children’s toothbrushing habits and diets, as well as to seeking dental services when needed. The main responsibility of fathers is to manage the financing of medical care. Therefore, we only looked at the relationship between maternal educational level and tooth decay, and we found statistically significant differences between maternal education and caries prevalence in both chi-squared and binary regression analysis (p<0.05). This is consistent with a number of other studies^2,30,34^ showing that the less educated the mother, the more likely the child is to develop caries. The reason may be that the mother’s educational level has an impact on her and her children’s oral health knowledge, oral care attitudes, and oral care behaviours. The higher the educational level of the mother, the richer the knowledge of oral health, the greater the demand for oral health care, and the better the oral health of the child.^6,24^

Next, although the effect of oral hygiene behaviours on the prevention of caries in some countries and studies is controversial,^3,16,21^ the present authors found a statistically significant difference between different oral hygiene behaviours and habits on caries prevalence (p<0.05). The higher the frequency of brushing and the longer the duration of brushing, the lower is the caries prevalence, which is consistent with most studies.^1,2,29^ However, Alraqiq et al^3^ and Kamran et al^16^ showed that there was no correlation between the occurrence of caries and the frequency of brushing, only a negative relationship between the brushing time and caries. In our chi-squared and regression analysis, we found correlations between brushing frequency and time and caries. At present, only scant literature exists on the relationship between dental floss use and caries, and the correlation is still unclear. However, only some studies^9,19^ have shown that flossing can prevent the occurrence of proximal carious lesions. The reason for this may be due to the fact that dental floss can remove the impacted food near the teeth and physically interfere with the adhesion of bacteria in the plaque biofilm, thereby reducing the amount of cariogenic bacteria and thus also the caries prevalence.^19^ A statistically significant relationship between dental flossing and caries prevalence was also found in our survey (p<0.05), and regression analysis showed that flossing was a protective factor (OR: 0.728, 95% CI: 0.553-0.994). Further longitudinal analysis is needed to clarify these results.

The relationship between dietary habits and tooth decay has been studied since the 1940s and is now a recognized and modifiable risk factor.^31,33^ However, the association with caries is different between countries, due to differences in dietary patterns, and even different within countries due to differences between different ethnic groups. In our study, although we only looked at some of the sugary, refined products that children in this age group like to consume, both the chi-squared tests and regression analysis showed that soft drinks, desserts, and especially confections (candy etc), were significantly positively correlated with caries prevalence (p<0.05). These results agree the results of many other studies.^10,24,28,33^ The higher the frequency of eating sugary foods, the greater is the incidence of caries. The mechanism for this is that when the plaque in the tooth biofilm decomposes carbohydrates, acidic substances – e.g., lactic acid and acetate – are released which change the pH of saliva and plaque. When the pH drops below the critical level of 5.5-5.7, a large amount of calcium and phosphate on the enamel surface is dissolved, which increases cariogenic potential.^12,28^

Our study has some advantages over other studies. First, a comprehensive and systematic analysis of the modifiable risk areas leading to the occurrence of caries was conducted, which goes beyond several previous studies in this field.^2,3,6,29^ Second, a larger sample size than many other studies was used to evaluate the current caries status of primary teeth. There have been few previous studies on the caries status of children aged 7-9 years, and such studies on northeast China are lacking. Finally, a comprehensive analysis of the carious lesions of each tooth of the primary teeth in the mouth was made, which has rarely been done in previous studies.

### Limitations

There are some limitations to our study, however. First, this is a cross-sectional study that cannot establish a causal relationship between risk factors and tooth decay, so further longitudinal studies are needed to obtain more accurate data. Second, risk factors were investigated in the form of questionnaires, which have a certain degree of recall bias. Third, the study was based on clinical examination only and did not incorporate dental radiographs. Finally, the sample of this study only involved decayed teeth in children aged 7-9 years, so it may not be generalisable the whole province or country.

## CONCLUSION

The caries prevalence in the primary teeth in Chinese children aged 7-9 years is fairly high, and our data show it to be related in particular to sociodemographic factors, oral health behaviours, and dietary habits. Therefore, we recommend multi-department and multi-center joint cooperation to develop prevention and treatment strategies to help children develop healthy oral hygiene and diet patterns as soon as possible, in order to help prevent caries and improve the quality of life.
